# Opioid Prescribing Behaviors — Prescription Behavior Surveillance System, 11 States, 2010–2016

**DOI:** 10.15585/mmwr.ss6901a1

**Published:** 2020-01-31

**Authors:** Gail K. Strickler, Peter W. Kreiner, John F. Halpin, Erin Doyle, Leonard J. Paulozzi

**Affiliations:** 1 Institute for Behavioral Health, Brandeis University, Waltham, MA; 2 Division of Unintentional Injury Prevention, National Center for Injury Prevention and Control, CDC; 3 Certified Technical Experts, Inc., Montgomery, AL

## Abstract

**Problem/Condition:**

In 2017, a total of 70,237 persons in the United States died from a drug overdose, and 67.8% of these deaths involved an opioid. Historically, the opioid overdose epidemic in the United States has been closely associated with a parallel increase in opioid prescribing and with widespread misuse of these medications. National and state policy makers have introduced multiple measures to attempt to assess and control the opioid overdose epidemic since 2010, including improvements in surveillance systems.

**Period Covered:**

2010–2016

**Description of System:**

The Prescription Behavior Surveillance System (PBSS) was created in 2011. Its goal was to track rates of prescribing of controlled substances and possible misuse of such drugs using data from selected state prescription drug monitoring programs (PDMP). PBSS data measure prescribing behaviors for prescription opioids using multiple measures calculated from PDMP data including 1) opioid prescribing, 2) average daily opioid dosage, 3) proportion of patients with daily opioid dosages ≥90 morphine milligram equivalents, 4) overlapping opioid prescriptions, 5) overlapping opioid and benzodiazepine prescriptions, and 6) multiple-provider episodes. For this analysis, PBSS data were available for 2010–2016 from 11 states representing approximately 38.0% of the U.S. population. Average quarterly percent changes (AQPC) in the rates of opioid prescribing and possible opioid misuse measures were calculated for each state.

**Results and Interpretation:**

Opioid prescribing rates declined in all 11 states during 2010–2016 (range: 14.9% to 33.0%). Daily dosage declined least (AQPC: -0.4%) in Idaho and Maine, and most (AQPC: -1.6%) in Florida. The percentage of patients with high daily dosage had AQPCs ranging from -0.4% in Idaho to -2.3% in Louisiana. Multiple-provider episode rates declined by at least 62% in the seven states with available data. Variations in trends across the 11 states might reflect differences in state policies and possible differential effects of similar policies.

**Public Health Actions:**

Use of PDMP data from individual states enables a more detailed examination of trends in opioid prescribing behaviors and indicators of possible misuse than is feasible with national commercially available prescription data. Comparison of opioid prescribing trends among states can be used to monitor the temporal association of national or state policy interventions and might help public health policymakers recognize changes in the use or possible misuse of controlled prescription drugs over time and allow for prompt intervention through amended or new opioid-related policies.

## Introduction

In 2017, a total of 70,237 persons in the United States died from a drug overdose, and 67.8% of these deaths involved an opioid recognized as a controlled^/^scheduled* substance by the federal government (*1*). Historically, the opioid overdose epidemic in the United States has been closely associated with a parallel increase in prescription opioid prescribing. The opioid prescribing rate in 2015 was three times that in 1999, yet the overall level of pain reported by persons in the United States did not change during this time (*2*–*4*). Various factors contributed to the increase in opioid prescribing, including changing attitudes toward the role of opioids in chronic pain management, calls for more liberal use of opioids by professional pain management societies, and state regulations that have encouraged the use of opioids for chronic pain management (*5*).

Although the general trend in opioid overdose deaths has been steadily upward over the past 20 years, deaths attributable to one subcategory of opioids, the natural and semisynthetic opioids, exhibited a brief period of decline during this time. In 2012, the death rate attributable to natural and semisynthetic opioids, which includes many of the most commonly prescribed opioids for chronic pain (e.g., oxycodone, hydrocodone, hydromorphone), decreased 8%, the first decline in this category since 1999 (*6*). However, by 2016, the death rate had once again risen above the level seen in 2011 (*6*). Reasons for this dip and rebound are not well understood. This period marked the introduction of mandates requiring physicians to check prescription drug monitoring programs (PDMPs) before prescribing controlled substances in selected states, the implementation of new state opioid prescribing guidelines, the development of reformulated medications designed to reduce misuse, and legislation regulating pain clinics (*7*). These policy changes make it particularly important to examine trends in opioid prescribing and possible patient misuse since 2010.

The Prescription Behavior Surveillance System (PBSS) is a public health surveillance system that uses PDMP data to monitor trends in prescribing behaviors for controlled substances at the state or county level (*8*). In 2010, PBSS began using PDMP data from participating states to report on a variety of indicators of prescribing behavior, including prescribing rates by patient age, sex, drug type, dose, and source of payment. Although data on clinical indication is not collected, the system tracks various controlled substance indicators of possible misuse, including cash payment for prescriptions and “multiple-provider episodes,” in which a person uses multiple prescribers and pharmacies within specified periods to obtain controlled substances. Several indicators of prescription opioid misuse have been associated with increased rates of overdose deaths among persons who use opioids (*9*) or with medical board actions against prescribing physicians (*10*). Other studies have suggested that inappropriate prescribing patterns (*11*) can lead to overuse of long-acting preparations of opioid medications that, in turn, are linked to increased risk for overdose death (*12*).

A previous study using PBSS data demonstrated that several indicators of controlled substance prescribing behavior varied substantially across eight states (*8*). The study used data from one calendar year (2013) and thus could not describe how trends among these indicators of controlled prescribing behaviors varied over time. This report presents PBSS data from 11 participating states to describe trends in prescription opioid prescribing behaviors during 2010–2016. Where feasible, key state and federal interventions (e.g., rescheduling of hydrocodone) were identified that might have affected these trends. Conducted routinely, such analysis might help public health policymakers recognize changes in the use or possible misuse of controlled prescription drugs that might explain or anticipate changes in prescription opioid overdose mortality on the state or national level and allow for prompt intervention through amended or new opioid-related policies.

## Methods

### Study Design

This report describes trends in measures of opioid prescribing behaviors for dispensed controlled substances during 2010–2016 in 11 states. This longitudinal descriptive analysis examined PDMP data that were obtained through PBSS at Brandeis University. Methods have been described in detail elsewhere (*8*). This study was reviewed and approved by the Brandeis University Institutional Review Board.

### Data Source and Data Collection

PBSS is a longitudinal, multistate database of de-identified data on prescriptions for scheduled drugs tracked by PDMPs in 12 states. The database was established in 2011 in response to the prescription drug overdose epidemic with funding from the CDC and the U.S. Food and Drug Administration, under Bureau of Justice Assistance administration, to serve as an early warning public health surveillance tool and an evaluation tool in relation to state and local policies and initiatives, such as prescriber educational initiatives. The database contains state PDMP data from 2010 (or the earliest available year) to 2016 and was updated on a quarterly basis. Data from 2017 were not analyzed in this study because of changes in patient record linkage algorithms used that year.

PBSS collects data on prescriptions of controlled substances to provide indicators of possible inappropriate medical use to both federal and state collaborators. PBSS has developed approximately 43 prescription behavior measures including prescription rates by drug class and individual drug, high daily opioid dosages (≥90 morphine milligram equivalents [MME]/day), average daily opioid dosage, overlapping opioid prescriptions and opioid-benzodiazepine prescriptions, multiple-provider episode (MPE) rates by drug schedule or class, payment sources, and indicators of possible inappropriate prescribing and dispensing. The database can detect changes in prescribing patterns earlier than other administrative health data (e.g., Medicaid claims data).

### Study Population

This report includes PDMP data from 11 of the 12 states participating in PBSS as of January 1, 2017 (California, Delaware, Florida, Idaho, Kentucky, Louisiana, Maine, Ohio, Texas, Virginia, and West Virginia), representing approximately 38.0% of the U.S. population in 2016 (*13*). At the time of this study, Washington was revising its patient record linkage algorithm and its PDMP data were not available for analysis. Complete data from 2010 through 2016 are presented for seven of the 11 PBSS states. For other PBSS states, PDMPs were either implemented after 2010 or have PDMP data retention policies that preclude obtaining earlier years of data. Thus, data from Delaware, Florida, and Idaho were only available for 2012–2016 and from Texas for 2015–2016.

### Measures

Six quarterly PBSS measures of opioid prescribing behaviors were restricted to prescriptions dispensed by in-state pharmacies to state residents. Buprenorphine products for substance use disorder treatment were excluded to allow comparisons to a recent national analysis that did not include buprenorphine for conditions other than pain (*2*) and because, during the surveillance period, there had been a substantial increase in buprenorphine for substance use disorder (*14*). Tramadol was excluded from analysis because it was only tracked by PDMPs after July 2014, when it became a scheduled drug.

Most measures are expressed as crude, population-based rates calculated from the most current state census information available from the U.S. Census Bureau^†^. The six measures examined were:

1. **Opioid prescribing rate**. Defined as the total number of Drug Enforcement Administration (DEA) Schedule II-V (CII-V) (*15*) opioid prescriptions dispensed in the quarter in the state per 1,000 state residents.
2. **Mean daily opioid dosage in MME.** Mean daily dosage is calculated for patients that have a CII-V opioid prescription in a given quarter and refers to MME per day prescribed (total number of MME prescribed divided by the total number of prescription days accounting for overlapping prescription days). Conversion factors have been published elsewhere (*16*).
3. **Percentage of patients with a high daily dosage of opioids**. Defined as the percentage of opioid-treated patients in the quarter with ≥90 MME per day prescribed for all CII-V opioid drugs used by the patient, calculated using the average daily MME for CII-V opioid drugs over the 3-month period.
4. **Percentage of opioid-treated days with overlapping CII-V opioid prescriptions**. Defined as the percentage of total opioid-treated days for all patients in the quarter with at least two overlapping CII-V opioid prescriptions.
5. **Percentage of opioid-treated days with overlapping benzodiazepine prescriptions.** Defined as the percentage of total opioid- and benzodiazepine-treated days for all patients in the quarter with overlapping CII-V opioid prescriptions and benzodiazepine prescriptions.
6. **MPE rates.** Defined as the number of instances per 100,000 state residents in which a patient filled CII-IV opioid prescriptions from five or more prescribers at five or more pharmacies during the previous 3 months. Reliable MPE data were not available for Idaho, Louisiana, Texas, and Virginia because PDMP data vendors changed during the surveillance period.

### Statistical Analyses

Analyses were conducted using SAS (version 9.4) to calculate the measures and Joinpoint segmented regression analysis software (version 4.5.0.1) to test whether changes in the measures over time were statistically significant (*17*). Joinpoint computes the average annual percent change, a summary measure of trends when the rates of change are not constant over a specified time interval (*18*). This report presents the average quarterly percent change (AQPC) because the time units of the data are calendar quarters, not years. A log transformation was applied and a maximum of five joinpoints were searched for using an overall alpha level of 0.05. The number of joinpoints varied from five for two measures (mean daily dosage in Ohio and percentage of days with overlapping opioid prescriptions in Maine) to zero for nine measures, including MPE rates in Delaware. The statistical significance of AQPCs were measured using 95% confidence intervals. When estimating AQPC, homoscedastic errors were assumed and autocorrelation was corrected for using an established method (*19*). Joinpoint analysis for Texas was not conducted because of limited data points in the 2 years of available data. The relative percentage change from the baseline quarter to the last quarter of available data also was calculated for each measure.

To highlight certain temporal associations, trend lines were graphed by state for each indicator with dates when notable national and state-level interventions addressing opioid prescribing behaviors had occurred. Figures include selected states with data from 2010–2016 that represented the highest, lowest, and approximate median rates for each measure. These states were selected to depict the range of values found across the states in the study. The full range of rate data for all 11 states in the study are presented (Supplementary Tables 1-6, https://stacks.cdc.gov/view/cdc/84092).

Prescribing measures were not age-adjusted because the primary focus was the examination of trends over time within each state rather than comparisons between states. However, a sensitivity analysis found that the age-adjusted rates for prescribing rates in four states (California, Delaware, Florida, and West Virginia) differed by less than 10%, on average, from the unadjusted rates.

## Results

**FIGURE 1:**
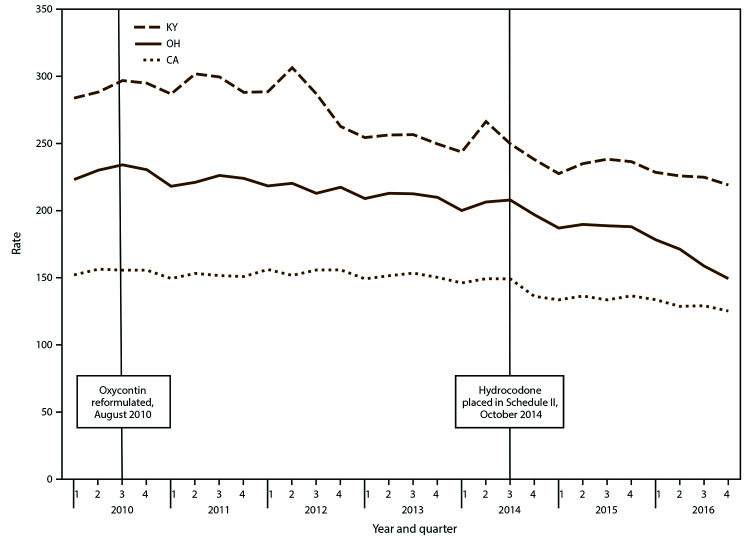
Opioid prescribing rate,* by selected states† and quarter — Prescription Behavior Surveillance System, 2010-2016^§,¶^ **Abbreviations:** CA = California; KY = Kentucky; OH = Ohio. * Opioid prescribing rate is defined as the total number of Drug Enforcement Administration Schedule II-V opioid prescriptions dispensed during the quarter in the state per 1,000 state residents. † Includes selected states with data during 2010–2016 that represented the highest, lowest, and middle rates for each measure. These states were selected to depict the range of values found across the states in the study. § The statistical significance of average quarterly percent changes was measured using 95% confidence intervals. The average quarterly percent change over the time period shown was statistically significant (p<0.05) for California, Kentucky, and Ohio. ¶ Number of Joinpoints: California = one, Kentucky = two, Ohio = two.

During 2010–2016, opioid prescribing rates declined in all PBSS states (Table) except Idaho and Louisiana. The quarterly opioid prescribing rate per 1,000 state residents in California decreased 17.7% (Table, Figure 1). The largest AQPC change occurred in Ohio (-1.6% per quarter and approximately −6.4% per year). The overall decline ranged from 14.9% to 33.0% from 2010 to 2016, excluding Texas because of insufficient data. From quarter three 2014 to quarter one 2015, opioid prescribing rates declined (range: 6.6%–13.8%), accounting for 45% of the overall decline in each of the eight states that showed statistically significant downward trends in opioid prescribing rates over the entire period.

**TABLE:** Trends in measures of opioid use and possible misuse,* by state^†^ — Prescription Behavior Surveillance System, United States, 2010–2016

State	Years	Opioid prescribing rate		Mean daily opioid dosage		Percent of patients with high daily dosage of opioid (≥90 MME)		Percent of opioid-treated days with overlapping opioid prescriptions episode rate		Percent of opioid-treated days with overlapping benzodiazepine prescriptions		Multiple-provider episode rate	
		Total % change	AQPC (95% CI)	Total % change	AQPC (95% CI)	Total % change	AQPC (95% CI)	Total % change	AQPC (95% CI)	Total % change	AQPC (95% CI)	Total % change	AQPC (95% CI)
CA	2010–16	**-17.7**	**-0.8 (-0.9 to 0.6)**	**-25.7**	**-1.2 (-1.6 to -0.8)**	**-32.5**	**-1.5 (-2.0 to -1.0)**	**-17.0**	**-0.7 (-0.8 to -0.6)**	**-9.5**	**-0.3 (-0.5 to −0.2)**	**-79.4**	**-6.0 (-8.1 to -3.8)**
DE	2012–16	**-18.6**	**-1.0 (-1.2 to -0.9)**	**-17.9**	**-1.0 (-1.2 to -0.7)**	**-25.0**	**-1.4 (-2.3 to -0.4)**	**24.5**	**1.1 (0.5 to 1.7)**	**-19.3**	**-1.2 (-1.5 to -0.9)**	**-75.5**	**-6.4 (-7.5 to −5.2)**
FL	2012–16	**-14.9**	**-0.8 (-1.4 to −0.2)**	**-27.4**	**-1.6 (-1.9 to -1.3)**	**-27.2**	**-1.4 (-1.9 to -1.1)**	-5.8	-0.3 (-1.0 to 0.3)	**-22.0**	**-1.3 (-2.3 to -0.4)**	**-76.6**	**-8.1 (-9.0 to -7.2)**
ID	2012–16	-3.4	-0.2 (-0.4 to 0.1)	**-7.4**	**-0.4 (-0.6 to -0.3)**	**-5.7**	**-0.4 (-0. To -0.3)**	4.9	0.3 (-0.1 to 0.7)	-2.8	-0.2 (-0.6 to 0.1)	—**^§^**	—**^§^**
KY	2010–16	**-22.8**	**-1.0 (-1.8 to -0.1)**	**-13.5**	**-0.5 (-0.7 to -0.3)**	**-38.7**	**-2.1 (-2.6 to -1.6)**	-19.9	-0.8 (-2.4 to 0.8)	**-18.0**	**-0.7 (-1.0 to -0.5)**	**-81.7**	**-6.1 (-8.1 to -4.1)**
LA	2010–16	-14.3	-0.5 (-0.9 to 0.0)	**-15.4**	**-0.7 (-0.9 to -0.5)**	**-43.9**	**-2.3 (-3.1 to -1.6)**	0.3	-0.1 (-0.9 to 0.7)	**-15.8**	**-0.7 (-1.4 to -0.1)**	—**^§^**	—**^§^**
ME	2010–16	**-25.0**	**-1.2 (-1.3 to -1.0)**	**-12.2**	**-0.4 (-0.5 to -0.3)**	-21.5	**-**1.1 (−2.3 to 0.2)	8.4	0.3 (-0.3 to 1.0)	−5.6	-0.1 (-0.7 to 0.6)	**-62.3**	**-4.1 (-4.8 to -3.5)**
OH	2010–16	**-33.0**	**-1.6 (-2.0 to-1.2)**	**-20.8**	**-0.9 (-1.1 to -0.6)**	**-43.3**	**-2.2 (-3.2 to -1.3)**	**-19.0**	**-0.9 (-1.1 to -0.7)**	**-21.6**	**-0.9 (-1.2 to -0.7)**	**-86.9**	**-7.3 (-9.8 to -4.8)**
TX	2015–16	11.3	—**^¶^**	-2.9	—**^¶^**	-12.8	—**^¶^**	9.7	—**^¶^**	−2.3	—**^¶^**	—**^§^**	—**^§^**
VA	2010–16	**-17.6**	**-0.7 (-1.0 to -0.4)**	**-12.2**	**-0.6 (-0.6 to -0.5)**	**-25.6**	**-1.8 (-2.8 to -0.9)**	6.5	0.0 (-0.3 to 0.3)	−4.1	0.0 (-0.7 to 0.7)	—**^§^**	—**^§^**
**WV**	**2010–16**	**-24.1**	**-1.1 (-1.7 to -0.6)**	**-12.2**	**-0.5 (-0.9 to -0.1)**	**-37.7**	**-2.0 (-2.7 to -1.3)**	**−6.2**	**−0.3 (-0.7 to 0.1)**	**-35.8**	**-1.7 (-2.3 to -1.1)**	**-94.8**	**-10.2 (-16.0 to -4.0)**

**Abbreviations:** AQPC = average quarterly percent change; MME = morphine milligram equivalent; CI = confidence interval.

* Opioid prescribing rate is defined as the total number of Drug Enforcement Administration (DEA) CII-V opioid prescriptions dispensed in the quarter in the state per 1,000 state residents. Mean daily opioid dosage is calculated for patients that have a DEA Schedule CII-V opioid prescription in a given quarter and refers to MME per day prescribed (total number of MME prescribed divided by the total number of prescription days accounting for overlapping prescription days). Percentage of patients with a high daily dosage of opioids is defined as the percentage of opioid-treated patients in the quarter with >90 MME per day prescribed for all DEA Schedule CII-V opioid drugs used by the patient, calculated using the average daily MME for Schedule CII-V opioid drugs over the 3-month period. Percentage of opioid-treated days with overlapping opioid prescriptions is defined as the percent of total opioid-treated days for all patients in the quarter with at least two overlapping DEA Schedule CII-V opioid prescriptions. Percentage of opioid-treated days with overlapping benzodiazepine prescriptions is defined as the percentage of total opioid- and benzodiazepine-treated days for all patients in the quarter with overlapping DEA Schedule CII-V opioid prescriptions and benzodiazepine prescriptions. Multiple-provider episode rate is defined as the number of instances in which a patient fills DEA Schedule CII-IV opioid prescriptions from five or more prescribers at five or more pharmacies in the previous 3 months per 100,000 state residents.

^†^ The statistical significance of AQPCs was measured using 95% confidence intervals. Changes significant at the 0.05 level are in bold.

^§^ Because of a change in data vendors during the period of study, trend analysis on multiple-provider episode rates in Idaho, Louisiana, Texas, and Virginia were not conducted.

^¶^ Data were not sufficiently reliable to permit trend estimates. In Texas, only eight quarters of data were available, which were not sufficient for Joinpoint analyses.

**FIGURE 2:**
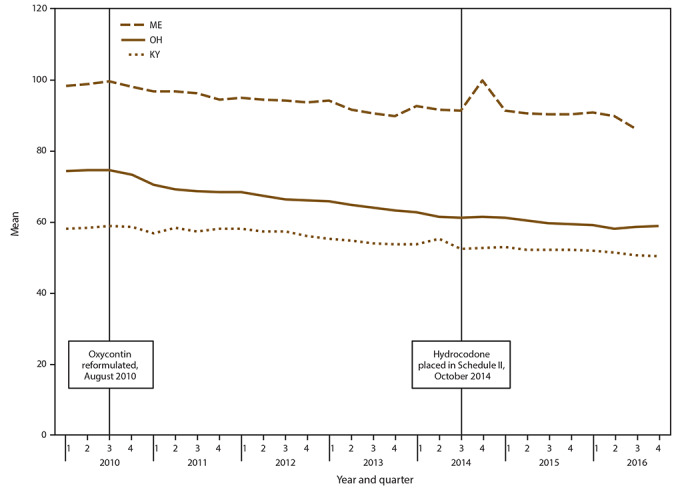
Mean daily opioid dosage* per patient in morphine milligram equivalent (MME), by selected states† and quarter — Prescription Behavior Surveillance System, 2010-2016^§,¶^ **Abbreviations:** KY = Kentucky; ME = Maine; OH = Ohio; MME = morphine milligram equivalent. * Mean daily opioid dosage is calculated for patients that have a Drug Enforcement Administration Schedule II-V opioid prescription during a given quarter and refers to MME per day prescribed (total number of MME prescribed divided by the total number of prescription days accounting for overlapping prescription days). † Includes selected states with data during 2010–2016 that represented the highest, lowest, and middle rates for each measure. These states were selected to depict the range of values found across the states in the study. § The statistical significance of average quarterly percent changes was measured using 95% confidence intervals. The average quarterly percent change over the time period shown was statistically significant (p<0.05) for Kentucky, Maine, and Ohio. ¶ Number of Joinpoints: Kentucky = two, Maine = none, Ohio = five.

In the first quarter of 2010, mean daily dosage ranged from 98.3 MME in Maine to 58.2 MME in Kentucky (Figure 2, Supplementary Table S2, https://stacks.cdc.gov/view/cdc/84092). During 2010–2016, mean daily opioid dosage declined significantly in all PBSS states except Texas, which did not have sufficient quarters of data for trend analyses (Table). The smallest decline in daily dosage (-7.4%) occurred in Idaho; the largest (-27.4%) in Florida. The largest percentage decline (-27.4%) was in Florida from quarter four 2011 to quarter four 2016. The largest AQPC also was in Florida (-1.6% per quarter). Florida and Maine experienced spikes in daily dosage during quarter four 2014 (Figure 2, Supplementary Table S2, https://stacks.cdc.gov/view/cdc/84092). For the latest quarter of data available, all states still had mean daily dosages >50 MME, and Delaware had a dosage >90 MME (Supplementary Table S2, https://stacks.cdc.gov/view/cdc/84092).

**FIGURE 3:**
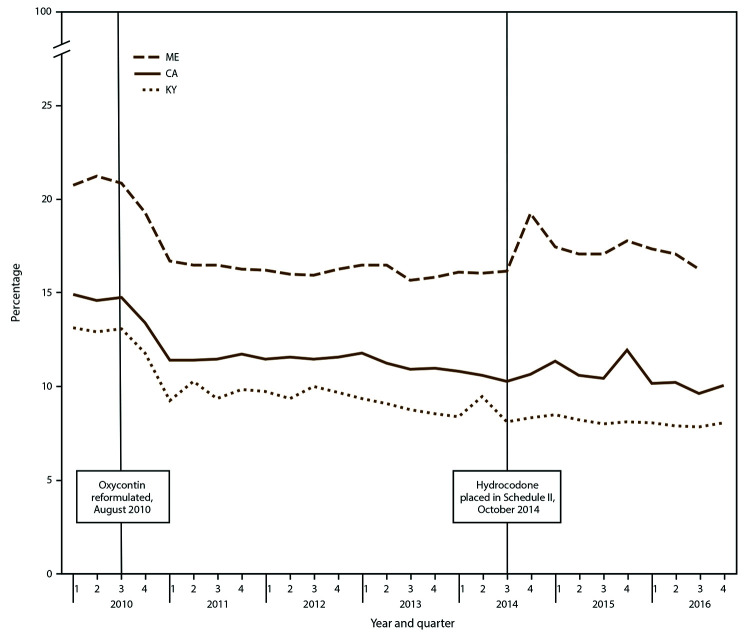
Percentage of patients receiving a high daily dosage of opioids* (>90 MME per day), by selected states† and quarter — Prescription Behavior Surveillance System, 2010-2016^§,¶^ **Abbreviations:** CA = California; KY = Kentucky; ME = Maine; MME = morphine milligram equivalent. * Percentage of patients with a high daily dosage of opioids is defined as the percentage of opioid-treated patients during the quarter with >90 MME per day prescribed for all Drug Enforcement Administration Schedule II-V (CII-V) opioid drugs used by the patient, calculated using the average daily MME for CII-V opioid drugs over the 3-month period. † Includes selected states with data during 2010–2016 that represented the highest, lowest, and middle rates for each measure. These states were selected to depict the range of values found across the states in the study. § The statistical significance of average quarterly percent changes was measured using 95% confidence intervals. The average quarterly percent change over the time period shown was statistically significant (p<0.05) for California and Kentucky but not Maine. ¶ Number of Joinpoints: California = one, Kentucky = one, Maine = three.

The percentage of patients with a high daily dosage (≥90 MME) of opioids had significant declines in AQPC in all PBSS states except Maine (Table, Figure 3). AQPCs ranged from -0.4% in Idaho to -2.3% in Louisiana. In quarter one 2010, the percentage of patients with high daily dosage ranged from 20.7% in Maine to 13.1% in Kentucky (Figure 3). Louisiana had the largest total percentage change (-43.9%) and the largest change in AQPC (-2.3%). All seven states with complete data from quarter three 2010 experienced substantial parallel declines in this measure from quarter three 2010 to quarter one 2011 (Supplementary Table S3, https://stacks.cdc.gov/view/cdc/84092). The four states with the highest daily percentage of high-dosage prescriptions (Delaware, Maine, Idaho, and Florida) had spikes in the percentage of ≥90 MME in the fourth quarter of 2014 (Supplementary Table S3, https://stacks.cdc.gov/view/cdc/84092). By quarter four 2016, Delaware still had 17.3% of patients on dosages ≥90 MME. PBSS data also indicated peaks in high-dosage rates in multiple states in the fourth quarter of 2014 (Supplementary Table S3, https://stacks.cdc.gov/view/cdc/84092).

**FIGURE 4:**
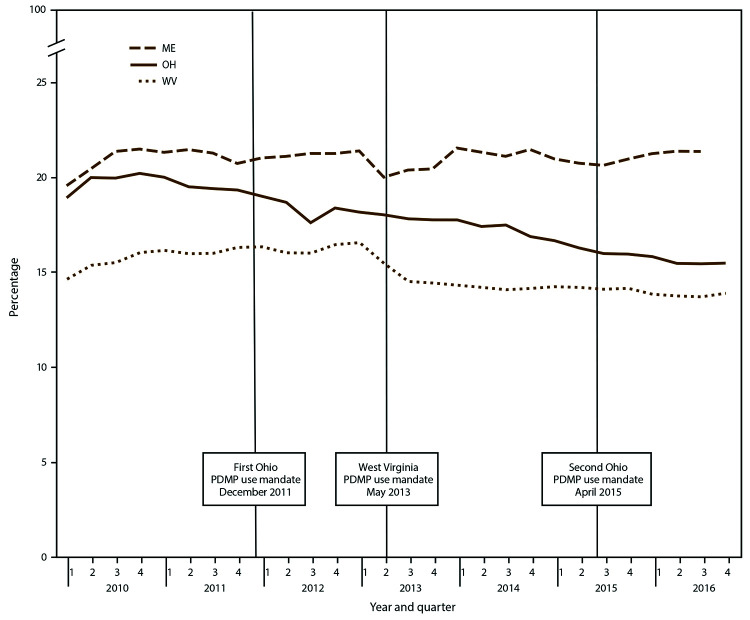
Percentage of opioid-treated days with overlapping opioid prescriptions,* by selected states† and quarter — Prescription Behavior Surveillance System, 2010-2016^§,¶^ **Abbreviations:** ME = Maine; OH = Ohio; WV = West Virginia; PDMP = prescription drug monitoring program. * Percentage of opioid-treated days with overlapping opioid prescriptions is defined as the percent of total opioid-treated days for all patients in the quarter with at least two overlapping Drug Enforcement Administration Schedule II-V opioid prescriptions. † Includes selected states with data during 2010–2016 that represented the highest, lowest, and middle rates for each measure. These states were selected to depict the range of values found across the states in the study. § The statistical significance of average quarterly percent changes was measured using 95% confidence intervals. The average quarterly percent change over the time period shown was statistically significant (p<0.05) for Ohio but not Maine or West Virginia. ¶ Number of Joinpoints: Maine = five, Ohio = four, West Virginia = three.

In the first quarter of 2010, 15%–20% of opioid-treated days overlapped (Figure 4). Compared with other measures, statistically significant changes in overlapping opioid prescriptions were less common over the study period (Table). The largest decline occurred in Ohio (-19.0%) with 18.9%–15.3% of opioid-treated days overlapping (Table, Figure 4). Delaware was the only state with a significant increase in this measure (24.5%), increasing from 23.5% to 29.3% from the beginning of 2012 to the end of 2016 (Table, Supplementary Table S4, https://stacks.cdc.gov/view/cdc/84092).

**FIGURE 5:**
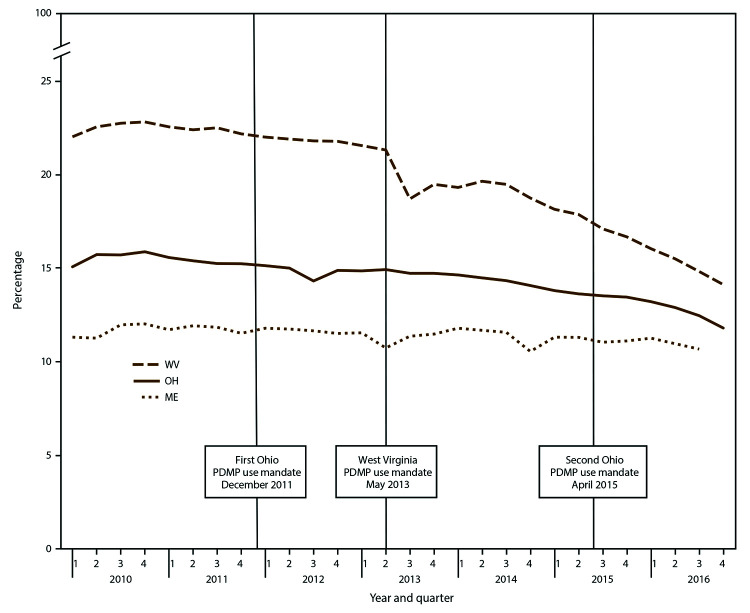
Percentage of opioid-treated days with overlapping benzodiazepine prescriptions,* by selected states† and quarter — Prescription Behavior Surveillance System, 2010-2016^§,¶^ **Abbreviations:** ME = Maine; OH = Ohio; WV = West Virginia; PDMP = prescription drug monitoring program. * Percentage of opioid-treated days with overlapping benzodiazepine prescriptions is defined as the percent of total opioid- and benzodiazepine-treated days for all patients during the quarter with overlapping Drug Enforcement Schedule II-V opioid prescriptions and benzodiazepine prescriptions. † Includes selected states with data during 2010–2016 that represented the highest, lowest, and middle rates for each measure. These states were selected to depict the range of values found across the states in the study. § The statistical significance of average quarterly percent changes was measured using 95% confidence intervals. The average quarterly percent change over the time period shown was statistically significant (p<0.05) for Ohio and West Virginia but not Maine. ¶ Number of Joinpoints: Maine = one, Ohio = four, West Virginia = three.

The percentage of overlapping opioid and benzodiazepine-treated days in quarter one 2010 ranged from 22.1% in West Virginia to 11.4% in Maine (Figure 5). West Virginia and Kentucky were the only states with percentages above 20% (Supplementary Table S5, https://stacks.cdc.gov/view/cdc/84092). The percentage decreased significantly in seven states, with the largest decrease (35.8%) in West Virginia (from 22.1% to 14.2% of treated days) (Table).

**FIGURE 6:**
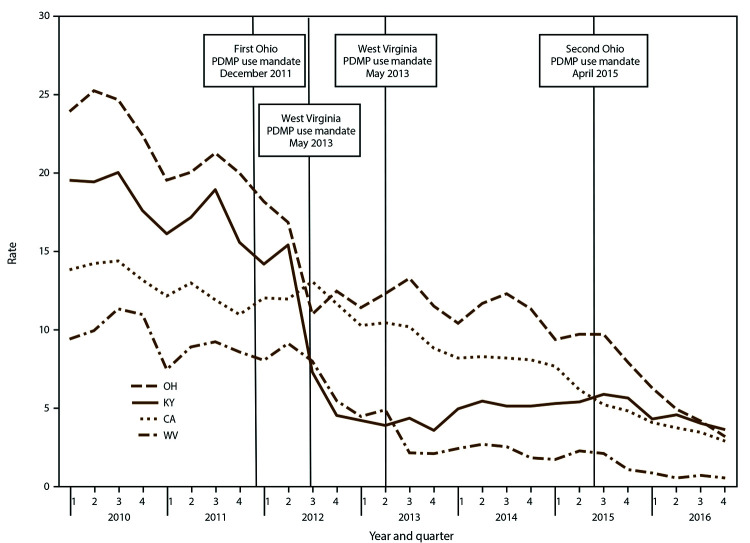
Multiple-provider episode rates,* by selected states† and quarter — Prescription Behavior Surveillance System, 2010-2016^§,¶^ **Abbreviations:** CA = California; KY = Kentucky; OH = Ohio; WV = West Virginia; PDMP = prescription drug monitoring program. * Multiple-provider episode (MPE) rate is defined as the number of instances in which a patient fills Drug Enforcement Administration Schedule II-IV opioid prescriptions from five or more prescribers at five or more pharmacies during the previous 3 months per 100,000 state residents. †Includes selected states with data during 2010–2016 that represented a range of values found across the states in the study with data available for this measure. § The statistical significance of average quarterly percent changes was measured using 95% confidence intervals. The average quarterly percent change over the time period shown was statistically significant (p<0.05) for California, Kentucky, Ohio and West Virginia. ¶ Number of Joinpoints: California = four, Kentucky = three, Ohio = none, West Virginia = four.

MPE rates varied among states in quarter one 2010, ranging from 4.3 per 100,000 state residents in Maine to 24.0 in Ohio (Figure 6, Supplementary Table 6, https://stacks.cdc.gov/view/cdc/84092). MPE rates decreased substantially during 2010–2016 in all states with available data, with the largest percentage declines for any of the six PBSS measures (Table). The largest total percentage decline occurred in West Virginia (94.8%) (Table). Kentucky experienced a sharp decline in 2012 from 15.4 per 100,000 in quarter two to 4.5 in quarter four (Figure 6).

## Discussion

The findings in this report indicate encouraging trends in controlled substance prescribing behaviors during 2010–2016 in 11 states that represent approximately 38.0% of the U.S. population and a range of geographic regions of the country. Over the years of available data, declines in state opioid prescribing rates ranged from 14.9% to 33.0% and declines in mean daily dosage ranged from 7.4% to 27.4%. The percentage of patients with high daily dosage declined, ranging from 5.7% to 43.9%. The percentage of days with overlapping opioid and benzodiazepine prescriptions declined, ranging from 4.1% to 35.8%. MPE rates declined by at least 62% in all states with available data. Overall, quarterly PBSS data revealed wide interstate variation in multiple measures and some sharp quarter-to-quarter changes.

PBSS measures of prescribing rates and possible opioid misuse are consistent with published national data (*2*,*20*). The overall opioid prescribing rate in this report, ranging from approximately 150 to 250 opioid prescriptions per 1,000 persons per quarter, is consistent with the national rate of approximately 80 opioid prescriptions on the basis of commercial data (*2*). Moreover, the declines in opioid prescribing rates observed in this study are similar to national trends, with national annual opioid prescribing rates remaining relatively constant during 2010–2012 followed by a 13% decrease during 2012–2015 (*2*).

Potential factors associated with the decline in prescribing include the introduction of a reformulated OxyContin with abuse-deterrent properties in August 2010 (*21*) and the rescheduling of hydrocodone combination products from DEA Schedule III to the more restrictive Schedule II (*15*) as of October 6, 2014 (Figure 1). Hydrocodone accounted for half of all opioid prescriptions in PBSS states in 2013 (*8*), and the rescheduling meant that hydrocodone could no long be prescribed with refills or by telephone. Although the trends since 2010 are encouraging, the national rate of opioid prescribing in 2015 remained triple the rate of opioid prescribing in 1999 (*2*).

National commercial data also indicate that the average daily dose per opioid prescription decreased nationally by 16.9% during 2010–2015 (*2*), which is consistent with the PBSS findings in this report (decline in average MME during 2012–2016 ranged from 7.4% to 27.4%). However, national data indicate that average daily MME per prescription declined from 58 MME in 2010 to 48 in 2015 (*2*), whereas mean daily dosage per patient in PBSS data ranged from 60 to 100 MME during 2010 and from 50 to 90 during 2016. The higher dosages observed from PBSS data are probably a result of PBSS adding the daily dosages on days with overlapping opioid prescriptions, which represent approximately 20% of all opioid-prescribed days as well as the exclusion of relatively low-dose tramadol prescriptions from this analysis. Therefore, PBSS data might provide a more accurate accounting of higher dosages prescribed compared with commercial data.

National annual high-dose opioid prescribing rates (≥90 MME) declined by 41% during 2010–2015, with the largest annual percentage decline occurring in 2011 (*2*). National data also indicate that approximately 10% of opioid prescriptions in 2015 were high-dose, which is similar to the percentage of patients with high daily dosage in PBSS data in 2015 for the large states of California and Texas but less than percentages in smaller states such as Delaware, Maine, and Idaho. The higher percentages in some PBSS states might reflect the ability of PBSS to combine dosages for overlapping opioid prescriptions.

PBSS data indicate that the high-dosage decline began in the first quarter of 2011, shortly after the reformulation of OxyContin in the third quarter of 2010. Patients with opioid dependence reportedly reduced use of OxyContin after reformulation (*22*), and an overall national decline in prescribing of extended-release oxycodone products occurred following reformulation (*21*). The data also indicate high-dosage increases in the fourth quarter of 2014. Hydrocodone combination products were rescheduled into Schedule II during October 2014, and the high-dosage increase might reflect a switch from hydrocodone to higher-strength extended-release opioids among persons who misuse prescription opioids (*23*) as well as a decline in the proportion of opioid prescriptions for hydrocodone, which tend to be lower dosage than oxycodone prescriptions (*8*). An overall decline in opioid prescribing following the rescheduling of hydrocodone has been reported (*24*), but the increase in the percentage of high-dose prescriptions has not been described previously.

A previous PBSS report documented interstate variation in opioid prescribing behaviors in 2013 (*8*), and this report builds on that observation by showing variation in trends in the risk measures examined across 11 states. Such variation in trends probably reflects a combination of different policies enacted in different states, possible differential effects of similar policies in different states, and different state contexts (*23*). For example, certain states enacted laws requiring prescribers to check PDMP before issuing a controlled substance prescription under a wide range of conditions (Kentucky in July 2012, Ohio in December 2011 and April 2015, and West Virginia in May 2013) (*25*). An analysis of the effects of these mandates found that their implementation was followed by decreases in the opioid prescribing rate, overlapping opioid prescription rate, overlapping opioid and benzodiazepine prescriptions rate, and MPE rate in Kentucky and Ohio but not in West Virginia (*26*).

Despite these favorable trends in national opioid prescribing rates during 2010–2015 and declines in opioid prescribing behavior indicators in PBSS states through 2016, opioid overdose deaths attributable to the most commonly prescribed opioids, the natural and semisynthetics (e.g., morphine and oxycodone), increased during 2010–2016 (*6*). Rates declined during 2011–2013 but then increased through 2016 (*1*). One possible explanation for this inconsistency is that changes in mortality lag behind changes in prescribing behaviors (*20*). Alternatively, since 2010, the trend in deaths related to these types of opioids has been driven by factors other than prescription opioid misuse rates, such as increasing mortality from heroin, which is frequently classified as morphine or found concomitantly with morphine postmortem (*24*), and a spike in deaths involving illicitly manufactured fentanyl combined with heroin and prescribed opioids since 2013 (*25*,*26*).

## Limitations

The findings in this report are subject to at least seven limitations. First, findings from the 11 states examined might not be generalizable to other states with differing populations of patients or prescribers or state laws and regulations governing the prescribing of controlled substances. Second, the analysis strategy and the timeframe for this study did not allow for rigorous testing of the impact of state or federal actions to improve opioid prescribing (e.g., the CDC Guideline for Prescribing Opioids for Chronic Pain) (*27*). Such changes are best interpreted using other study designs (e.g., difference-in-differences analyses) (*7**,**28**,**29*), which are beyond the scope of this study. Third, states use different methods to link patient records in their PDMP data, and it is unknown how these methods differ in their accuracy of identifying unique patients. Fourth, direct comparison of the prescribing rates across states must be done with care because of differences between states in their patient age distributions and the association between opioid prescribing and age (*8*). However, the sensitivity analysis in this report suggests that age differences probably have a small effect on such comparisons. Moreover, interstate age differences should have no effect on the changes seen within individual states over short time periods. Fifth, information regarding the medical indication for use of opioids is not available in PBSS data, so it was not possible to determine whether the prescriptions were appropriately prescribed based on the condition being treated. Sixth, limitations in data collection in some states prevented inclusion of certain data in all analyses. Finally, the data exhibit period-to-period variation, or noise, around the underlying trends. Joinpoint analysis assumes that the amount of this noise is similar in different periods, but this requires sufficient data over time to confirm. The post hoc examination of the data over the seven-year period available suggests that this assumption is, in fact, warranted, and that the significant trends seen in the data are valid. Even then, changes are difficult to attribute to any specific intervention.

##  Conclusion

Surveillance data are critical for addressing the epidemic of drug overdoses in the United States. Administrative data collected by state prescription drug monitoring programs, which is available in 49 states (*30*), can be repurposed to track the known risk factors that contribute to prescription opioid-related deaths on a population basis. State health departments, boards of pharmacy, and boards of medicine can use PDMP data to track trends in opioid prescribing behaviors and indicators of possible misuse.

PDMP data collected by PBSS indicate that steady progress is being made in reducing the use and possible misuse of prescription-controlled substances in the United States. However, some persons who were misusing prescription opioids might have transitioned to heroin or illicitly manufactured fentanyl, a change that has made the drug overdose epidemic and associated overdose rates more complex (*31*). Because the opioid overdose epidemic began with increased deaths and treatment admissions related to opioid analgesics in the late 1990s (*32*,*33*), initiatives to address overprescribing might eventually result in fewer persons misusing either prescription or illicit drugs. Reduction in overprescribing opioids might lead ultimately to a decrease in overall overdose deaths.

## Footnotes

* The Controlled Substances Act places all substances that were in some manner regulated under existing federal law into one of five schedules.  This placement is based on the substance’s medical use, potential for abuse, and safety or dependence liability.  More information is available in Title 21 United States Code. Controlled Substances Act.
